# Effects of Vagus Nerve Stimulation following Corpus Callosotomy for Patients with Drug-Resistant Epilepsy

**DOI:** 10.3390/brainsci11111395

**Published:** 2021-10-23

**Authors:** Keisuke Hatano, Ayataka Fujimoto, Takamichi Yamamoto, Hideo Enoki, Tohru Okanishi

**Affiliations:** 1 Comprehensive Epilepsy Center, Seirei Hamamatsu General Hospital, 2-12-12 Sumiyoshi, Nakaku, Hamamatsu 430-8558, Shizuoka, Japan; hatakenosuke@gmail.com (K.H.); taka-yamamd@sis.seirei.or.jp (T.Y.); enokih.neuropediatr@gmail.com (H.E.); t.okanishi@tottori-u.ac.jp (T.O.); 2 Department of Neurosurgery, Seirei Hamamatsu General Hospital, 2-12-12 Sumiyoshi, Nakaku, Hamamatsu 430-8558, Shizuoka, Japan

**Keywords:** vagus nerve stimulation (VNS), corpus callosotomy (CC), drug-resistant epilepsy

## Abstract

Objective: The effectiveness of vagus nerve stimulation (VNS) for residual seizures after corpus callosotomy (CC) has not yet been fully investigated. We hypothesized that seizure control would be improved by VNS after CC. The purpose of this study was to compare seizure frequency between patients with implantation of a VNS generator (post-VNS group) or without VNS (non-post-VNS group) following CC. Methods: We retrospectively reviewed patients who underwent CC between January 2009 and May 2019 in our institution. We evaluated proportions of ≥50% reduction in seizure frequency (responders) and seizure reduction rate 1 and 2 years after VNS. To investigate factors related to responders, uni- and multivariate logistic regression analyses were performed regarding age, number of anti-seizure medications (ASMs), addition of novel ASMs (levetiracetam, lacosamide or perampanel), and post-VNS or non-post-VNS status. Results: Thirteen post-VNS patients and 24 non-post-VNS patients were analyzed in this study. Responder rate at 1 year after VNS differed significantly between the post-VNS group (53.9%) and non-post-VNS group (12.5%, *p* = 0.017). Number of ASMs at the time of CC and post-VNS were significantly associated with responders in univariate analyses (odds ratio [OR] 0.34, 95% confidence interval [CI] 0.13–0.88, *p* = 0.025 and OR 8.2, 95%CI 1.6–41.6, *p* = 0.011, respectively), whereas age, sex, seizure frequency, and addition of novel ASMs were not. In multivariate analysis, the presence of VNS procedures after CC was the only factor favorably associated with responder status (OR 82.2, 95%CI 1.55–4355.7, *p* = 0.03). Conclusions: VNS therapy after CC may increase the proportion of responders independent of the addition of novel ASMs.

## 1. Introduction

Palliative techniques such as corpus callosotomy (CC) and vagus nerve stimulation (VNS) can be considered for patients with drug-resistant epilepsy who are not candidates for resective surgery. Satisfactory efficacy of both techniques for drug-resistant epilepsy has been demonstrated, with the proportion of a ≥50% reduction in seizure frequency reported as 40–70% for VNS [1,2,3,4,5,6] and 63–79% for CC [1,2,3,7]. On the one hand, VNS is less invasive and carries a low risk of severe complications such as epidural or subdural hematoma and disconnection syndrome, which are complications of CC [2,8,9,10]. On the other hand, CC is more likely to improve seizure frequency or severity than VNS, particularly for drop attacks or epileptic spasms [9,10,11,12]. The selection of these two techniques appears to depend on seizure type, surgical risk, and the wishes of the patient and caregivers, although no universally accepted indications have been determined. The exact mechanisms underlying the effects of VNS remain unclear [13]. However, since a difference exists between VNS, which involves the plasticity of the brain [14,15], and corpus callosotomy, which involves severing the corpus callosum, the mechanisms are considered to differ. Better control of seizures may thus be obtained by applying a combination of the two methods rather than either VNS or CC.

Patients may undergo CC first and achieve no favorable improvement of seizures, but may then receive VNS as additional surgery. However, only two reports have described outcomes of additional VNS therapy after CC, suggesting that the implantation of VNS after CC for patients with Lennox-Gastaut syndrome (LGS) induced a ≥50% reduction in seizure frequency in 57–60% of patients [5,16].

However, no case-control studies appear to have compared patients who received VNS implantation after CC with those who underwent CC alone. Since novel anti-seizure medications (ASMs) have appeared one after another over the last two decades, the effects of novel ASMs need to be taken into account to more accurately compare the outcomes of VNS and CC. In Japan, levetiracetam (LEV), perampanel (PER), and lacosamide (LCM) became available clinically from September 2010, May 2016, and July 2016, respectively. Case-control studies comparing outcomes from patients with VNS to those for patients without VNS after CC without bias from these new ASMs appear to be required.

The present study retrospectively reviewed patients who did and did not receive implantation of VNS after CC, investigating the efficacy of additional VNS after CC as the purpose of this study. We hypothesized that the addition of VNS therapy after CC could induce more favorable improvement of seizures than CC without addition.

## 2. Methods

### 2.1. Participants and Study Design

This study was a single-site, retrospective investigation conducted in Japan. The Ethics Committee of Seirei Hamamatsu General Hospital approved the protocol for this study (approval no. 3768), which was performed in accordance with the principles of the Declaration of Helsinki. Participants in this study were identified via a retrospective study of patients treated at the Comprehensive Epilepsy Center, Seirei Hamamatsu General Hospital. Written informed consent was obtained from all patients. Written informed consent for publication of data pertaining to participants under the age of 19 years was obtained from the patients’ guardians.

We reviewed the data for patients with refractory epilepsy who underwent CC between January 2009 and May 2019 in our institution. We then compared patients implanted with a VNS generator after CC (post-VNS group) to patients not implanted with a VNS generator after CC (non-post-VNS group). The inclusion criterion was patients with poor outcomes after CC {International League Against Epilepsy (ILAE) classification 4–6} [17]. Exclusion criteria were as follows: (1) Patients who had experienced complications in the CC such as cerebral infarction/hemorrhage, subdural/epidural hematoma, surgical site infection, or abscess; (2) cases with follow-up duration <24 months; (3) patients with excellent outcomes after CC (ILAE classification 1–3 [17]); (4) patients who underwent other epilepsy surgeries such as focus resection; or (5) patients who requested to opt out of participation in the study. We excluded patients with ILAE classification 1–3 after CC, because we did not usually propose VNS therapy to these patients. In the post-VNS group, we also excluded those patients in whom the VNS generator had been removed.

As demographic data, we reviewed age at epilepsy onset and at the time of CC, sex, epilepsy syndromes such as West syndrome or LGS, number and type of ASMs at the time of CC and VNS therapy, seizure type at the time of CC, seizure frequency at the time of CC, extent of CC (total callosotomy or anterior callosotomy), and model of VNS generator implanted. We divided seizure frequency into daily (≥1 seizure/day), weekly (≥1 seizure/week but <1 seizure/day), monthly (≥1 seizure/month but <1 seizure/week), and yearly (≥1 seizure/year but <1 seizure/month).

### 2.2. VNS Therapy

We performed VNS therapies using only a 103 Demipulse VNS generator (VNS Therapy^®^ System; Cyberonics, Houston, TX, USA) until August 2014 and also using the 105 Aspire HC (VNS Therapy^®^ System; Cyberonics) after August 2014 and the 106 Aspire SR (VNS Therapy^®^ System; LivaNova, Houston, TX, USA) after August 2018. The device was activated 2–3 weeks after implantation of VNS. We increased the current output (0.25 mA at once) or duty cycle (<10% at once) at each visit until optimal seizure reduction was obtained.

### 2.3. Primary Outcome Measurement

**Figure 1:**
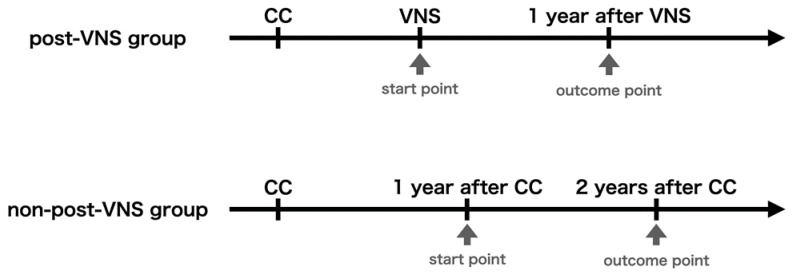
We defined the start point as the time before vagus nerve stimulation (VNS) in the post-VNS group and as 1 year after corpus callosotomy (CC) in the non-post-VNS group. Outcome points were defined as 1 and 2 years after the start point in both groups (this figure only shows the outcome point at 1 year after start point). In the non-post-VNS group, the outcome point 1 year after the start point corresponds to the time 2 years after CC.

Patients were classified as responders when they showed a ≥50% reduction in seizure frequency and as non-responders for a <50% reduction. We evaluated the proportion of responders and the seizure reduction rate from start point to outcome point. The start point was defined as the time before VNS therapy in the post-VNS group, and as 1 year after CC in the non-post-VNS group (Figure 1). Outcome points were defined as 1 and 2 years after the start point in both groups. Given that we usually observed patients who underwent CC for 6–12 months and proposed VNS therapy if CC was ineffective after observation, we used 1 year after CC as the start point for the non-post-VNS group.

### 2.4. Secondary Outcome Measurements

We also investigated predictive factors associated with responders and the following data were compared between responders and non-responders: age, sex, number of ASMs, addition of novel ASMs (LEV, LCM, or PER) from start point to outcome point, and post-VNS or non-post-VNS group. To investigate the presence or absence of addition of novel ASMs, we reviewed the number and type of ASMs at both start and outcome points.

### 2.5. Statistical Analyses

We used the *χ*^2^ test to compare categorical variables, or Fisher’s exact test when the sample size was small. Student’s *t*-test or the Mann–Whitney *U* test was used to compare continuous variables. Two-sided *p*-values < 0.05 were considered statistically significant.

To show factors associated with responder status, we used logistic regression analysis with adjustment for age, sex, number of ASMs, addition of novel ASMs from start point to outcome point, and post-VNS or non-post-VNS group.

In this study, VNS treatment affected prognosis in both uni- and multivariate analyses. However, since the sample size was not sufficiently large, statistical power was lacking for multivariate analysis.

All statistical analyses were performed using Stata/SE 14.0 (StataCorp LP, College Station, TX, USA).

## 3. Results

We performed CC for 84 patients in our institution between January 2009 and May 2019. We identified 16 patients who had undergone VNS therapy following CC in our institution and 68 patients who had not undergone VNS therapy after CC. Among these, 13 patients in the post-VNS group and 24 patients in the non-post-VNS group met the criteria. The mean interval from CC to VNS in the post-VNS group was 26.9 ± 34.9 months.

### 3.1. Clinical Profiles of Patients

Eleven of 13 patients (84.6%) in the post-VNS group and 13 of 24 patients (54.2%) in the non-post-VNS group were male. Median age at onset of epilepsy was 2 years in the post-VNS group and 1.5 years in the non-post-VNS group. Median age at the time of CC was 8 years in the post-VNS group and 18 years in the non-post-VNS group. The number of ASMs just before CC was significantly lower in the post-VNS group (3.0 ± 0.20) than in the non-post-VNS group (3.7 ± 0.22; *p* = 0.04).

Seizure frequency before CC did not differ significantly between post-VNS and non-post-VNS groups (daily, weekly, monthly, and yearly: 10, 2, 1, and 0 in the post-VNS group vs. 20, 2, 2, and 0 in the non-post-VNS group; *p* = 0.82), but seizure frequency at the start point differed significantly between groups (daily, weekly, monthly, and yearly: 11, 0, 0, and 2 in the post-VNS group vs. 12, 5, 6, and 1 in the non-post-VNS group; *p* = 0.018).

Extent of CC, number of ASMs at the start point, presence or absence of West syndrome or LGS, seizure type, and addition of novel ASMs from the start point to outcome point were similar between groups. The VNS generators implanted in the post-VNS group were an Aspire SR in 1 patient, Aspire HC in 5 patients, and Demipulse VNS in 7 patients (Table 1).

### 3.2. Primary Outcome Measures: 50% Responder Rate and Seizure Reduction Rate

**Figure 2:**
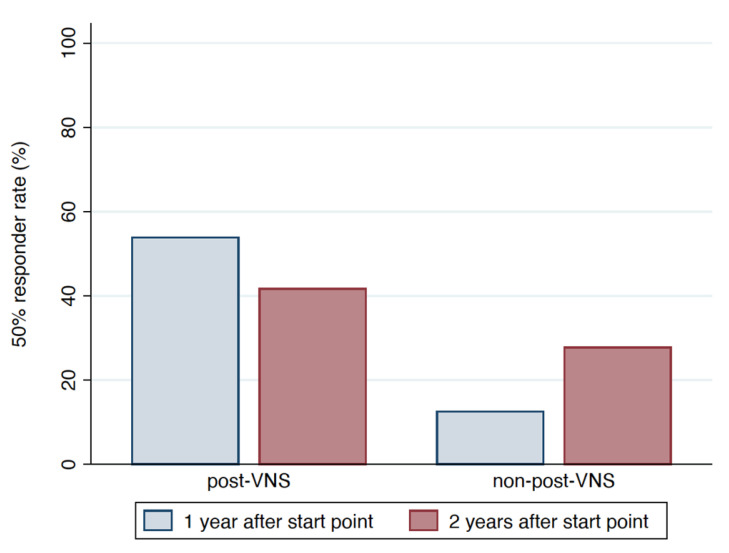
Histogram illustrating the frequency of achieving responder status (≥50% reduction in seizure frequency) at 1 year after start point was 53.85% in the post-VNS group and 12.5% in the non-post-VNS group (blue, *p* = 0.017, Fisher’s exact test). The frequency of achieving responder status at 2 years after the start point was 41.67% in the post-VNS group and 27.78% in the non-post-VNS group (red, *p* = 0.461, Fisher’s exact test).

**Figure 3:**
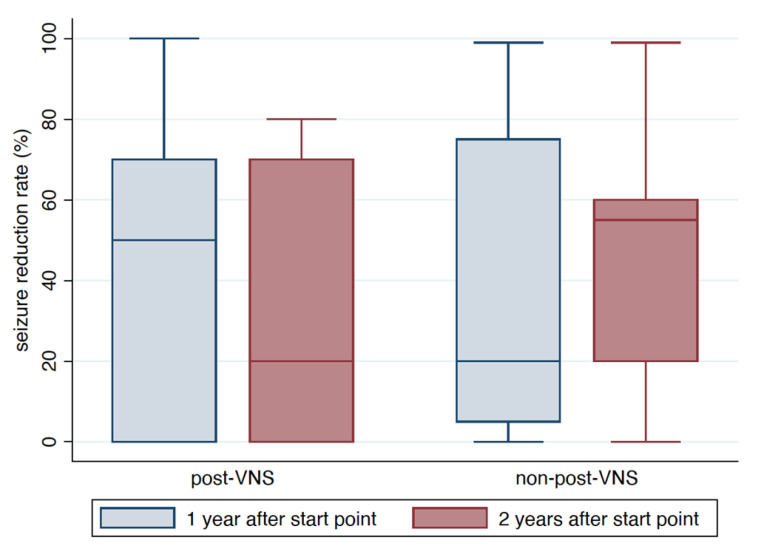
Boxplot illustrating seizure reduction rate. The seizure reduction rate in the post-VNS group was 50% (IQR 0–75%) and 20% (IQR 0–70%) at 1 and 2 years after the start point, respectively (*p* = 0.4943). Seizure reduction rate in the non-post-VNS group was 20% (IQR 2.5–77.5%) and 55% (IQR 17.5–60%) at 1 and 2 years after the start point, respectively (*p* = 0.0268).

Figure 2 and Figure 3 show the 50% responder rate and seizure reduction rate, respectively.

The proportion of responders from start point to 1 year after the start point differed significantly between the post-VNS and non-post-VNS groups (53.85% [7/13 cases] vs. 12.5% [3/24 cases], *p* = 0.017). The proportion of responders from start point to 2 years after the start point was similar between groups (41.67% [5/12 cases] in post-VNS group vs. 27.78% [5/18 cases] in the non-post-VNS group, *p* = 0.461).

The seizure reduction rate in the post-VNS group decreased from 1 year to 2 years after VNS, but this difference was not significant (median 50%; interquartile range [IQR] 0–75% at 1 year after VNS vs. median 20%, IQR 0–70% at 2 years after VNS, *p* = 0.4943). The seizure reduction rate in the non-post-VNS group increased significantly from 1 year to 2 years after start point (median 20%, IQR 2.5–77.5% at 1 year after start point vs. median 55%, IQR 17.5–60%) at 2 years after start point, *p* = 0.0268).

### 3.3. Secondary Outcome Measures: Factors Associated with Responders

Preoperative predictors related to responders were analyzed using uni- and multivariate logistic regression analyses (Table 2). The number of ASMs at the time of CC and the presence or absence of post-VNS correlated significantly with responders in univariate analysis (odds ratio [OR] 0.34, 95% confidence interval [95%CI] 0.13–0.88, *p* = 0.025 and OR 8.2, 95%CI 1.60–41.6, *p* = 0.011, respectively). Age at time of CC, sex, seizure frequency at start point, and addition of novel ASMs were not significantly associated with responder status. In multivariate analysis, the presence or absence of post-VNS was the only factor significantly correlated with responders (OR 82.2, 95%CI 1.55–4355.7, *p* = 0.03).

## 4. Discussion

This study investigated the effects of additional VNS after CC, comparing patients who received VNS after CC with those who did not. This study revealed that additional VNS after CC significantly increased the proportion of patients showing a ≥50% reduction in seizure frequency at 1 year after VNS regardless of the addition of novel ASMs. The results of this retrospective case-control study supported our hypothesis that the addition of VNS therapy after CC could induce more favorable improvement of seizures than CC without additional VNS.

Two non-comparative studies have investigated the outcomes of VNS after CC for patients with LGS showed favorable results with a responder rate at 1 year after VNS of 57–60% [5,16]. Similar studies reviewing the efficacy of VNS for patients with prior epilepsy surgery (not necessarily CC) have suggested a responder rate of 52.5% and a median seizure reduction rate of 45.7% at 1 year after VNS [6,18]. The current study revealed responder rates at 1 and 2 years after adding VNS to CC were 53.85% and 41.67%, respectively. This result appeared similar to the findings of previous reports. As a result, additional VNS following CC for drug-resistant epilepsy may achieve a ≥50% reduction in seizure frequency at 1 year after VNS in approximately 50% of cases.

In this study, the responder rate at 2 years after VNS was less than at 1 year after VNS, although some authors have documented VNS efficacy improving over time [6,8,19]. Kawai et al. also showed in a sub-analysis that the responder rate at 3 years after VNS was significantly lower in patients with prior epilepsy surgery than in those without prior epilepsy surgery (52.7% and 64.3% respectively, *p* = 0.033), although responder rates at 1 and 2 years after VNS were similar in both groups. The efficacy of VNS may not improve over time for patients who have already undergone other epilepsy surgeries, including CC, and thus may not obtain favorable improvement of seizures, because such patients are likely to have more refractory epilepsy.

We compared patients who underwent VNS after CC (post-VNS) with those who did not (non-post-VNS) and used multivariate analysis to eliminate the seizure-reducing effects of novel ASMs. Many reports have examined the efficacy of novel ASMs for refractory epilepsy. The ≥50% reduction rate was reported as 39–58% with LEV [20,21], 35.7–67% with LCM [22,23,24], and 31–68% with PER [25,26,27,28] from 6 months to 1 year after starting administration for patients with refractory epilepsy. In this study, the responder rate at 1 year after start point was only 12.5% in the non-post-VNS group. This low responder rate appears to be associated with the fact that only 3 patients (25%) added novel ASM in the non-post-VNS group.

On the other hand, the responder rate at 1 year after VNS in the post-VNS group was 53.85%, significantly higher than that in the non-post VNS group. In both groups, we had prescribed optimal ASMs for each outpatient. Furthermore, multivariate logistic regression analysis revealed that the addition of VNS after CC was the only factor significantly correlating with responder status, whereas addition of novel ASMs was not. The present study therefore revealed that VNS therapy for patients who had no favorable improvement of seizures after CC could significantly increase the frequency of achieving responder status, independent of the addition of novel ASMs such as LEV, LCM, or PER.

Several limitations to the present study must be acknowledged. First, this study was a retrospective observational study at a single facility. Patients with more refractory epilepsy might have been included in the non-post-VNS group rather than the post-VNS group, although patient characteristics other than seizure frequency at the start point were similar between groups. Second, this study included only a small sample. The small number of patients with addition of novel ASMs may have been associated with the result that the addition of novel ASMs could not significantly increase the frequency of responders according to logistic regression analysis. Third, half of the non-post VNS group had already received VNS therapy before CC. This may have led to an increase in patients with more intractable epilepsy in non-post VNS. To address these potential sources of bias, prospective registry studies or larger population-based studies across multiple institutions are needed to increase the generalizability of the study results.

## 5. Conclusions

Addition of VNS therapy to patients who could not obtain adequate seizure control after CC may increase the proportion of responders showing a ≥50% reduction in seizure frequency independent of the addition of novel ASMs. Given the present results, prospective and multicenter studies appear warranted.

## Figures and Tables

**Table 1:** Patient Characteristics.

	Post-VNS, *n* = 13	Non-Post-VNS, *n* = 24	*p* Value
sex-male	11 (84.62%)	13 (54.17%)	0.083
Age			
at onset	2 (0–7.5)	1.5 (0–8)	0.5859
at the time of CC	8 (6–16.5)	18 (4.25–35.5)	0.3003
ASM no. at the time of CC	3 ± 0.1961161	3.708333 ± 0.2209922	0.0412
Total CC	8 (61.54%)	13 (54.17%)	0.666
West/Lennox-Gastaut syndrome	9 (69.23%)	9 (37.5%)	0.091
Seizure frequency at the time of CC			0.816
daily	10 (76.92%)	20 (83.33%)	
weekly	2 (15.38%)	2 (8.33%)	
monthly	1 (7.69%)	2 (8.33%)	
Seizure type at the time of CC			
Tonic seizure	7 (53.85%)	10 (58.82%)	0.785
GTC	3 (23.08%)	3 (18.75%)	1
spasm	9 (69.23%)	5 (31.25%)	0.066
myoclonic seizure	1 (7.69%)	1 (6.25%)	1
atypical absence	4 (30.77%)	4 (25%)	1
focal seizure	2 (15.38%)	5 (31.25%)	0.41
drop seizure	4 (30.77%)	6 (37.5%)	1
ASM no. at the start point	3.307692 ± 0.2861015	3.85 ± 0.2643264	0.1863
Seizure frequency at the start point			0.018
daily	11 (84.62%)	12 (50%)	
weekly	0 (0%)	5 (20.83%)	
monthly	0 (0%)	6 (25%)	
yearly	2 (15.38%)	1 (4.17%)	
New AED add (from start point to 1y)			
LEV	1 (8.33%)	0 (0%)	0.364
LCM	2 (16.67%)	2 (9.52%)	0.61
PER	0 (0%)	3 (14.29%)	0.284
LEV/LCM/PER	3 (25%)	5 (23.81%)	1
VNS before CC	-	12 (50%)	

Data are given as median (IQR), mean ± standard deviation (SD), or number (%). ASM, anti-seizure medication; CC, corpus callosotomy; IQR, interquartile range; LCM, lacosamide; LEV, levetiracetam; PER, perampanel; SD, standard deviation; VNS, vagus nerve stimulation.

**Table 2:** Analyses of predictive factors associated with responder rate 1 year after the start point.

	Univariate Analysis			Multivariate Analysis	
	Odds Ratio	95%CI	*p* Value	Odds Ratio	95%CI
Age (CC)	0.993074	0.9442873–1.044381	0.787	1.062129	0.9551765–1.181056
sex	0.7285714	0.1528159–3.473567	0.691	4.21656	0.1307365–136.0044
ASM no. (CC)	0.3441279	0.1351764–0.8760701	0.025	0.1930513	0.0317687–1.173127
Seizure frequency (start point)	0.7273487	0.431977–1.224686	0.231	2.091066	0.4527822–9.657089
post-VNS	8.166667	1.602285–41.62458	0.011	82.1642	1.549928–4355.658
LEV_add	1				
LCM_add	1.277778	0.1119308–14.58683	0.844		
PER_add	1				
LEV or LCM or PER_add	1.333333	0.4333738–10.28394	0.355	0.4258999	0.0102008–17.78198

ASM, anti-seizure medication; CC, corpus callosotomy; LCM, lacosamide; LEV, levetiracetam; PER, perampanel.

## Data Availability

Considering our participants’ privacy, no data is publicly available in this study.

## Abbreviations

ASM	anti-seizure medication
CC	corpus callosotomy
IQR	interquartile range
LCM	lacosamide
LEV	levetiracetam
LGS	Lennox-Gastaut syndrome
OR	odds ratio
PER	perampanel
VNS	vagus nerve stimulation
